# Papillary renal neoplasm with reverse polarity: a case report and literature review

**DOI:** 10.3389/fonc.2025.1598738

**Published:** 2025-09-08

**Authors:** Diego Gonzalez, Kris Kokoneshi, Sam Kwon, Ryan Thomas Mathews, Ryan Michael Antar, Maher Ali, Abiye Kassa, Michael Whalen

**Affiliations:** ^1^ Department of Urology, George Washington University School of Medicine, Washington, DC, United States; ^2^ Department of Pathology, George Washington University School of Medicine, Washington, DC, United States

**Keywords:** oncology, renal cell carcinoma, renal cancer, papillary renal neoplasm with reverse polarity (PRNRP), renal neoplasm

## Abstract

**Introduction:**

Papillary renal neoplasm with reverse polarity (PRNRP) is a rare subtype of papillary renal cell carcinoma (RCC) with unique morphology, molecular features, and good prognosis. Given its rarity, with less than 100 reported cases, further characterization is needed to enhance diagnostic accuracy and inform management strategies.

**Case presentation:**

We report the case of a 59-year-old African American female with an incidentally discovered 2.1 cm left renal mass on imaging. The patient has a medical history of hypertension, asthma, hyperlipidemia, vertigo, depression/anxiety, and prediabetes. Further evaluation via contrast-enhanced CT confirmed an enhancing renal mass without evidence of metastasis. She underwent a robotic-assisted partial nephrectomy, and postoperative pathology confirmed PRNRP with pT1aNxMxR0 staging and ISUP nuclear grade 1. Immunohistochemical analysis demonstrated positive staining for GATA3, CK7, and EMA, and Vimentin and negative for AMACR and CA IX. Molecular profiling revealed a KRAS mutation, a key feature of PRNRP. Postoperative recovery was uneventful aside from transient vertigo, and no further treatment was required.

**Conclusion:**

This case reinforces the distinct morphological and molecular profile of PRNRP, distinguishing it from other papillary RCC subtypes. The indolent behavior, absence of metastatic cases, and characteristic molecular profiling and immunohistochemical markers highlight the importance of accurate classification for optimal patient management. Furthermore, the PD-L1 positivity observed in this case raises potential implications for immune checkpoint therapy, an area warranting further investigation. As PRNRP is recently classified, continued study is essential to refine diagnostic, therapeutic, and surveillance strategies for this rare renal neoplasm.

## Introduction

Papillary RCC is the second most common type of kidney cancer, behind clear cell carcinoma. Beginning in 1997, papillary RCC was separated into subtypes of type I and type II papillary RCC based on unique morphologic features (1). Yet, almost half of cases fail to meet every morphologic feature of these two types. Thus in 2022 the WHO Classification of Tumours of the Urinary System and Male Genital Organs recognized a variant of papillary RCC known as Papillary renal neoplasm with reverse polarity (PRNRP) to describe a small, well-circumscribed neoplasm that is morphologically distinct from both type I and II papillary RCC. It was first characterized in 18 patients in 2019 by Al-Obaidy et al. Type I and II PRCC are separated based on their histological findings, along with the fact that type II is considered high grade disease with a less favorable diagnosis (2). PRNRP is characterized by its recurrent (85%) KRAS gene mutation and unique nuclear arrangement, described histologically as a monolayer epithelial lining consisting of eosinophilic cells with granular cytoplasm and round nuclei, of low grade and apical location (3). PRNRP is universally characterized as low WHO/ISUP nuclear grade. When considering a diagnosis of PRNRP, it is important to consider the distinct morphological and IHC features that separate it from other tumors which may be part of the differential, including clear cell papillary renal cell carcinoma (CCPRCC), oncocytic papillary renal cell carcinoma (OPRCC), and papillary renal cell carcinoma type 1 (PRCC1) (4).There have only been ~100 published cases of PRNRP since Al-Obaidy et al. first characterized the subtype in 2019 (5), thus there is a need for further description of classification–both from histological and molecular perspectives–, management, and surveillance schedule.

## Case presentation

We report the case of a 59-year-old African American female who presented with a 2.1cm left renal mass found incidentally on imaging. The patient has a medical history of chronic intermittent vertigo, overactive bladder, anxiety/depression, hypertension (HTN), asthma, obesity, hyperlipidemia (HLD), vitamin D deficiency, and prediabetes (preDM). The patient has a surgical history of hysterectomy and tubal ligation. The patient reported chronic low back pain following a rear-ended vehicle accident, prompting an MRI of the lumbar spine in January 2024. After an incidental left renal mass was detected on the MRI, she underwent CT of the abdomen and pelvis with contrast for further evaluation that demonstrated a 2 x 1.8 cm left interpolar enhancing renal mass without retroperitoneal or pelvic lymphadenopathy is seen.

The patient opted to proceed with left robotic partial nephrectomy which was successfully performed with enucleoresection with warm ischemia time of 23 minutes. Postoperative course was complicated by onset of vertigo which was managed allowing for hospital discharge on postoperative day #3. Final pathology revealed pT1aNxMxR0 papillary renal neoplasm with reverse polarity, ISUP nuclear grade 1. No additional treatment was administered following the surgery.

## Pathology

Serial sectioning revealed a tan-brown, solid, soft, well-defined, hemorrhagic mass measuring 2 x 1.9 x1.5 cm, abutting the capsule and measuring 0.1 cm from the resection margin.

**Figure 1:**
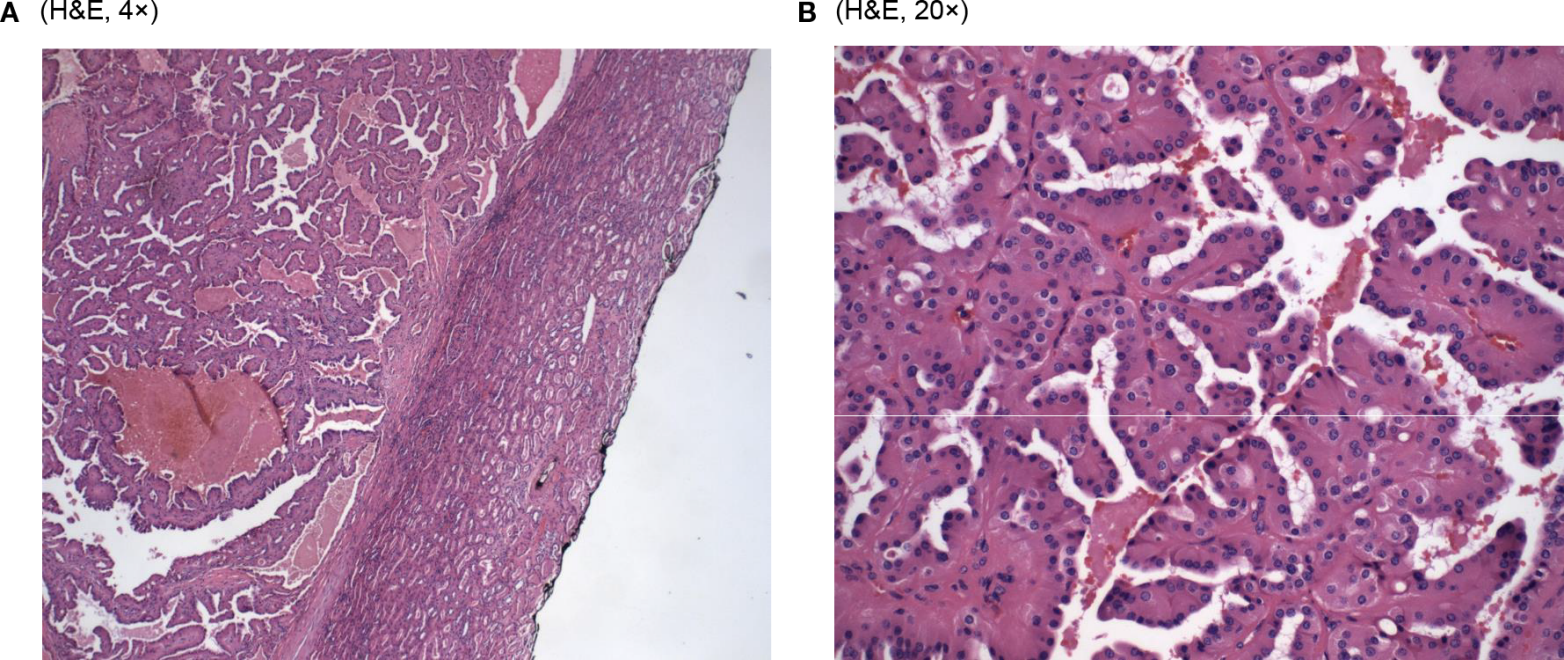
**(A)** (H&E, 4×). **(B)** (H&E, 20×).

Microscopic examination of the resected specimen revealed cystic and branching papillary tumor cells (**Figure 1A**). The tumor cells were predominantly papillary architecture composed of eosinophilic tumor cells with finely granular, oncocytic cytoplasm and low-grade nuclei (WHO/ISUP grade 1). Unlike classic papillary renal cell carcinoma, the nuclei were apically positioned (**Figure 1B**). No tumor necrosis, lymphatic or vascular invasion was identified. The tumor was identified as a well-differentiated histologic grade of G1, with nucleoli absent at 400x magnification.

**Figure 2:**
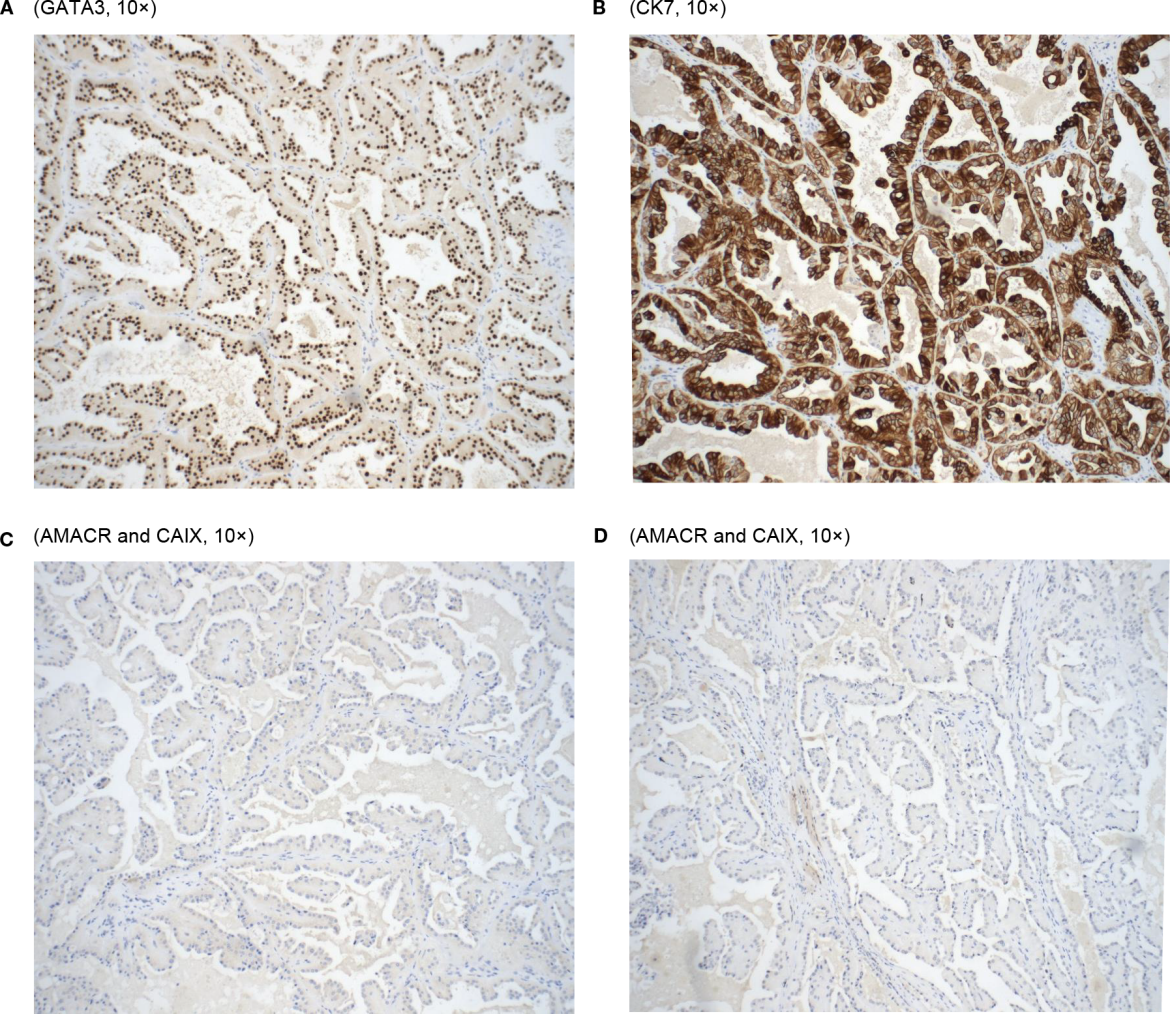
**(A)** (GATA3, 10×). **(B)** (CK7, 10×). **(C)** (AMACR and CAIX, 10×). **(D)** (AMACR and CAIX, 10×).

Immunohistochemically, the tumor cells were positive for GATA-3, CK7, and EMA (apical membranous) while negative for AMACR and CA IX (**Figure 2**). Vimentin was focally positive in the basal and basolateral portion of the tumor cells. The postoperative pathological diagnosis confirmed PRNRP with a clinical stage of pT1a.

## Discussion

As of 2022, the WHO considers PRNRP to be a variant of papillary RCC with a specific molecular driver alteration of a KRAS mutation (6). While Al-Obaidy et al. first coined the term papillary renal neoplasm with reverse polarity in 2019 that is still used today, PRNRP has potentially been described in the literature since 2003; Allory et al. used the term “oncocytoid-type renal cell carcinoma” in four patients with variant histology, describing the tumor cells as medium to large sized and eosinophilic with round regular nuclei. The authors also note the favorable clinical outcomes for these patients, fitting in line with the indolent course described in most cases of PRNRP (7). More distinct methods to diagnose and characterize PRNRP were not described in the literature until 2017, with Saleeb et al. conducting a histologic, IHC, and molecular analysis of papillary RCC subtypes. Here, the authors describe 6 cases with the distinct apically arranged nuclei of PRNRP, along with the oncocytic eosinophilic cytoplasm and round nuclei. In IHC analysis, this subtype, which the authors named “oncocytic low grade papillary renal cell carcinoma”, was positive for GATA3, negative for CA IX, and strongly and diffusely positive for ABCC2 (similarly to PRCC type II).

In contrast, PRCC type II was negative for GATA3, positive for CA IX (exhibiting a perinuclear dot-like Golgi pattern instead of its normal cell membrane localization), and showed a gain of chromosomes 5 and 8q. In gene set enrichment analysis, 43% of significantly enriched pathways were common between this subtype and PRCC type II. Finally, cluster analysis showed a distinct molecular cluster of this subtype (8). In sum, while bearing some similarities to PRCC type II, PRNRP still sets itself apart by way of its unique histological characteristics and gene expression profile.

In Al-Obaidy et al.’s paper coining PRNRP, the authors compare 18 cases of PRNRP with 30 controls of PRCC type I and II. Through histological comparison in their study, they found key diagnostic markers that could differentiate PRNRP from PRCC type I and II. The four criteria are: 1) papillary or tubulopapillary architecture of tumors, 2) these tumors are covered or lined by a single layer of eosinophilic cells with finely granular cytoplasm, 3) tumor cells have apically positioned nuclei with inconspicuous nucleoli, and 4) absence of intracellular hemosiderin, mitotic figures, and necrosis. Despite these four criteria discussed above, Yang et al. do report 11 cases of PRNRP of which hemosiderin deposits were identified in 6 tumors and multifocal/patchy necrosis identified in 5 tumors (9). This apparent contradiction highlights the evolving understanding of PRNRP and underscores the need for broader case series and consensus on its defining features, given its relatively recent characterization as a distinct renal tumor subtype. The diagnosis is further aided by comparing the immunohistological staining between PRNRP and PRCC type I, the authors found a significant difference in staining (p<0.05) between the groups for GATA3, L1CAM, epithelial membrane antigen, vimentin, and alpha-methylacyl-CoA-racemase (AMACR/p504s). GATA3 specifically was a key marker that was present in all PRNRP tumors but was negative when staining in PRCC type I and present in only one tumor of PRCC type II. In the FISH analysis conducted within the study, Al-Obaidy et al. cited a significant difference in chromosome 17 trisomy (p<0.03) between PRNRP and PRCC type 1 with it being more prevalent in type I tumors. Similarly, they noted a significant difference in chromosome Y deletions (P<0.01) with them being far more prevalent in PRCC type I and II.

Another study conducted by Chang et al. looked to investigate the association between PRNRP and the morphologically similar type D renal papillary adenoma analog. The immunohistological analysis in this study showcased the same relationship of GATA3 strongly staining in all but one case of PRNRP where it stained moderately (80%). The histological diagnostic criteria they decided upon were similar to Al-Obaidy et al. however it added one new criterion with the presence of focal or diffuse stromal hyalinization within tumor cells (10).

Looking at the microscopic examinations, our tumor sample did show the papillary architecture of the tumor with eosinophilic cells with granular cytoplasm and low-grade apically located nuclei (**Figure 1B**). As found in both studies, our patient’s tumor cells stained positively for GATA3. In addition, it stained positively for CK7 coinciding with the results of PRNRP tumors in Al-Obaidy et al. However, our sample varied when comparing the staining of Vimentin (+) and AMACR (-) to the tumor samples found within the Al-Obaidy study. When analyzing our sample with the Chang criteria, it was in accordance with all the criteria except we did not note any focal or diffuse stromal hyalinization. Overall, histopathological evaluation and immunohistochemical analysis are the cornerstone techniques for the accurate diagnosis of PRNRP.

Analysis of the TEMPUS molecular profiling demonstrated the presence of a KRAS gene mutation in our tumor sample, which is considered a key molecular feature of PRNRP (3). Moreover, consistent with the findings of Al-Obaidy et al., our tumor sample exhibited a low tumor mutational burden (2.6 mutations per megabase) (11). Interestingly, we found our tumor sample to be programmed death-ligand 1 (PD-L1) positive with a combined positive score (CPS) of 50%. This finding provides novel insights into the immune milieu of PRNRP as there is a paucity of literature exploring the immune microenvironment of PRNRP. The PD-L1 positivity with a CPS of 50% suggests an increased level of PD-L1 expression, which may indicate a favorable response to PD-L1/PD-1 checkpoint inhibitor immunotherapy. Yet, the immunogenic characteristics and therapeutic implications of immunotherapy in PRNRP remain largely unexplored, warranting further studies to investigate these areas.

As stated previously, a KRAS mutation is one defining molecular characteristic of PRNRP. In a review of 93 cases in the literature, Wei et al. reported that a KRAS mutation was found in 85% (73/86) of cases tested and a missense mutation in p.G12V was the most common type. (36/67 cases, 54%). The authors also reported that CK7 is expressed in 99% of cases (87/88), while GATA3 positivity is observed in all tested cases (68/68). Vimentin expression is rare, noted in 4% (3/72) of cases, with weak or focal positivity in an additional 7% (5/72). AMACR shows positivity in 36% (32/88) of cases, with weak or focal staining in 20% (18/88) (5). The immunophenotypic findings in our study align with the majority of these previously reported cases, including strong positivity for CK7 and GATA3. However, unlike prior studies where AMACR positivity is observed in a significant subset of cases (36% positive, 20% weakly/focally positive), AMACR was negative in our case. Additionally, our case demonstrated vimentin positivity, a feature rarely reported in the literature (4–7% of cases).

To date, no cases of locally advanced PRNRP (pT3-4) have been reported in the literature. All reported cases have been assigned a pathological stage of pT1-2, indicating that the tumors were confined to the kidney. The largest reported tumor diameter is 9.5 cm; however, the mean tumor diameter across all reported cases generally remained below 3 cm [**Table 1**]. Besides the low-grade characteristics of PRNRP, studies investigating the radiographic appearance of PRNRP on computed tomography (CT) imaging suggest that PRNRP may have distinguishing features from papillary RCC. Many studies have noted that the non-contrast CT of PRNRP displays high attenuation and an inhomogeneous enhancement pattern (16, 32, 36), which differ from the typical hypoenhancement of papillary RCC on contrast-phase CT.

**Table 1:** Prior case reports/series of papillary renal neoplasm with reverse polarity.

Author	Institution Type	Year	# of patients	PN (%)	RN (%)	Mean Tumor Diameter (cm)	Maximum Tumor Diameter (cm)	ISUP/WHO Grade	Pathological Stage	Margin Status
Wei (5)	Single	2022	7	n/a	n/a	3.1	8.5	1-2	pT1a, pT2a	n/a
Satturwar (12)	Single	2023	1	n/a	n/a	1.1	1.1	1	n/a	n/a
Xing (13)	Single	2023	1	100	0	3	3	1	n/a	n/a
Wang (14)	Single	2022	1	100	0	1.7	1.7	n/a	n/a	n/a
Li (15)	Single	2023	1	n/a	n/a	2	2	1	pT1a	n/a
Tu (16)	Single	2023	1	100	0	2.6	2.6	n/a	pT1	n/a
Zhang (17)	Single	2021	1	n/a	n/a	1.8	1.8	n/a	n/a	n/a
Yuzhi (18)	Single	2022	2	100	0	2.1	2.5	2	pT1a	n/a
Wu (19)	Single	2024	2	50	0	1.1	1.1	1	n/a	Negative
Lee (20)	Single	2020	1	0	100	1.2	1.2	1-2	pT1a	n/a
Al-Obaidy (3)	Multi	2019	18	50	39	1.6	3	1-2	pT1a	n/a
Tong (21)	Multi	2020	13	85	15	2.4	4.5	1-2	pT1a, pT1b	n/a
Zhou (22)	Multi	2020	7	71	29	2.9	8	1-2	pT1	n/a
Kiyozawa (23)	Multi	2021	14	n/a	n/a	2	5	1-2	pT1aN0M0, pT1bN0M0	n//a
Pivovarcikova (24)	Multi	2021	2	n/a	n/a	2	2.1	2	pT1a	n//a
Kim (25)	Multi	2020	30	97	3	1.8	5.8	1-3	pT1a	n/a
Conde-Ferreiros (2)	Single	2022	6	n/a	n/a	0.8	1.3	1	n/a	n/a
Wang (4)	Single	2022	15	93	7	2.2	3.5	1-2	pT1a	n/a
Kim (26)	Single	2023	43	91	9	1.6	n/a	1-2	pT1N0M0	n/a
Chen (27)	Single	2022	1	n/a	n/a	1.5	1.5	n/a	n/a	n/a
Yang (9)	Multi	2022	11	82	18	2.7	6	1-2	pT1aN0M0, pT1bN0M0	n/a
Shen (28)	Multi	2022	16	75	n/a	2.6	9.5	1-2	pT1a, pT1b, pT2a	n/a
Han (29)	Single	2024	9	89	11	2.2	3.7	1	pT1a	n/a
Shao (30)	Single	2024	2	100	0	2	2.5	n/a	n/a	n/a
Liu (31)	Single	2022	20	85	15	2	6	1-2	pT1a	n/a
Wen (32)	Multi	2024	26	92	8	2.2	4.9	n/a	pT1a, pT1b	n/a
Castillo (33)	Multi	2024	8	n/a	n/a	1.3	5.1	1-2	pT1	n/a
Chang (10)	Multi	2021	8	50	50	1.6	4.3	1-2	pT1-2	n/a
Al-Obaidy (34)	Single	2022	50	32	66	0.6	3	1-2	pT1a	n/a
Nemours (35)	Multi	2024	10	40	60	2.1	4	1	pT1a, pT1b	n/a
Lee (36)	Single	2024	31	n/a	n/a	1.7	n/a	n/a	n/a	n/a
Park (37)	Multi	2024	10	n/a	n/a	n/a	n/a	1-2	n/a	n/a

PN = partial nephrectomy; RN = radical nephrectomy; n/a = not applicable.

Although the majority of PRNRP diagnoses were made via pathological examination of specimens following either partial or radical nephrectomy, three studies documented PRNRP diagnosis through renal biopsy (3, 19, 34). In Wu et al., the authors describe a case in which a prospective PRNRP diagnosis was made using fine needle aspiration and core needle biopsy. Similarly, Al-Obaidy et al. noted cases of PRNRP being diagnosed on renal biopsy in both of their studies. Given PRNRP’s generally indolent behavior and the absence of metastatic cases, further studies on imaging and biopsy diagnostics may hold valuable potential for optimal patient management. This could translate to improved patient selection for conservative approaches, such as active surveillance (19, 36), prior to resorting to surgical intervention.

Currently, there is a lack of literature describing whether PRNRP has any hereditary predisposition. There has also been little discussion on the optimal treatment approach for PRNRP, likely due to its recent recognition in the literature and its generally indolent clinical behavior. As a result, most studies focus primarily on molecular, histologic, and immunophenotype characterization of this RCC subtype rather than treatment course. Thus, clinicians follow standard practice guidelines, recommending surgical resection as the optimal treatment for any non-metastatic solid renal mass, with a preference for minimally invasive techniques whenever possible (11). For localized tumors like our patients, surgeons may offer partial or radical nephrectomy. Partial nephrectomy is preferred when anatomically and technically feasible, as preservation of normal renal parenchyma significantly improves renal function and quality of life in comparison to radical nephrectomy (38). Alternative treatment options for renal masses smaller than 3 cm include thermal ablation, cryoablation, and radiofrequency ablation. As of now, no cases of PRNRP treated with ablative techniques have been reported in the literature. Finally, active surveillance is another option for patients. This is usually reserved for tumors smaller than 2 cm and when the anticipated benefits of intervention do not surpass those of active surveillance, with patients fully informed about the risks associated with surveillance (39). In all reported cases to date, PRNRP has been treated with either radical or partial nephrectomy. Further studies with long-term follow-up are needed to evaluate the efficacy of this approach and to consider potential adjuvant therapies if recurrence or metastasis is observed. As of now, no reports of recurrence, metastasis, or tumor-related death have come to light in the currently limited time available for follow-up post resection (5).

In conclusion, PRNRP is a recently characterized low-grade renal tumor that distinguishes itself from traditional papillary RCC types I and II through its unique histologic features and distinct gene expression profile. Its indolent clinical behavior is supported by the absence of mitotic figures and necrosis, as well as the lack of reported cases of recurrence or metastasis. Further research is essential to better understand its molecular origins, establish long-term outcomes, and develop evidence-based guidelines for management and surveillance, particularly in cases with atypical presentations.

## Data Availability

The original contributions presented in the study are included in the article/supplementary material, further inquiries can be directed to the corresponding author.
